# Urine Sampling Protocol Recommendations for Reliable Determination of Total Urinary Luteinizing Hormone Immunoreactivity in the Pediatric Population

**DOI:** 10.3390/children10121919

**Published:** 2023-12-12

**Authors:** And Demir, Adem Aydin, Atilla Büyükgebiz

**Affiliations:** 1 Pediatric Research Center, New Children’s Hospital, University of Helsinki and Helsinki University Hospital, 00290 Helsinki, Finland; 2 Department of Pediatrics, Faculty of Medicine, Dokuz Eylül University, Izmir 35340, Türkiye; 3 Department of Pediatrics, Division of Pediatric Endocrinology, Demiroğlu Bilim University, Istanbul 34394, Türkiye

**Keywords:** luteinizing hormone, day-to-day variation, urine, sampling protocol, correction, onset of puberty

## Abstract

This study investigates day-to-day variations in urinary luteinizing hormone (U-LH) concentrations in children, focusing on potential minimization or correction methods. 95 children and adolescents (51 boys, 44 girls, ages 5–17) provided daytime and evening urine samples for U-LH determinations over three consecutive days. No consistent day-to-day differences in U-LH levels were observed, although random variations, particularly in adolescents aged 13 or older, were noted. The net inter-assay CV% for U-LH changes over three days showed high variability, averaging 24.6% to 28.0% for boys and 21.6% to 27.3% for girls, independent of sex, collection time, or U-LH level. To reliably determine total urinary luteinizing hormone immunoreactivity in the pediatric population, it is advisable to collect multiple first-morning voided samples for at least three consecutive days as an interim solution, pending the development of a standardized protocol or correction method for varying urine composition. Strict adherence, especially for adolescents aged 13 or older, is vital.

## 1. Introduction

Nearly 50 years ago, timed urinary FSH and LH measurements were proposed as a non-invasive method to assess gonadotropin function in children [1,2,3]. In 1970, Rifkind et al. successfully quantified urinary FSH and LH using radioimmunoassay (RIA) [4]. Methods involving extraction and concentration were developed to increase sensitivity, and the use of timed urine samples was introduced to improve accuracy [5]. Despite these enhancements, the gonadotropin concentrations in prepubertal urine continued to fall below the detection limits of the RIA, even after applying these processing techniques. To overcome this limitation, researchers have assessed total gonadotropin excretion by analyzing urine samples collected over both 24-h and shorter periods [3,5,6]. A significant discovery by Bourguignon et al. in 1980 identified a morning rise in gonadotropin secretion at the beginning of puberty, indicating that gonadotropin excretion exhibits a circadian rhythm. This finding was based on the analysis of unextracted urine samples, which were gathered as timed fractions from 24-h urine collections [7].

Girard et al. recommended the first-morning voided (FMV) urine as an alternative to the 24-h output, citing the latter’s cumbersomeness and potential unreliability in children [8]. Following the development of ultrasensitive immunofluorometric assays, which provided high sensitivity [9,10], Stenman et al. showed that this method could also be applied to detect gonadotropins in urine samples [11]. Studies in the 1990s further investigated this promising non-invasive testing approach for urinary gonadotropins [12,13,14,15]. More recent research has demonstrated that the concentration of LH in FMV urine is a sensitive indicator of pulsatile pituitary gonadotropin secretion, indicating its clinical utility in the evaluation of puberty and its disorders [16,17,18,19,20,21,22,23,24,25,26]. In the meantime, the challenge of invasiveness continued to be a concern, even with the recognition of early morning S-LH as a dependable indicator of central puberty onset in the 2010s, as evidenced by its first documentation in 2013 by Lee et al. and later references in numerous reviews and guidelines, presenting it as a less invasive option compared to the standard GnRH-stimulation test [27,28,29,30,31,32,33,34].

Our previous studies on the central onset of puberty have documented that an increase in FMV urinary LH (U-LH) levels precedes the physical onset of puberty [26] and suggested that FMV urinary gonadotropin measurements might be an alternative to the invasive GnRH-stimulation test, traditionally the gold standard for diagnosing pubertal disorders [24]. Additionally, our most recent studies have uncovered novel insights into the physiology of central pubertal development, revealing that biochemical puberty onset occurs at approximately the same ages in both boys and girls [35] and that FMV U-LH measurements can predict the imminent onset of central puberty in girls within a year [36]. In our subsequent study, we investigated the alternative approach of assessing overnight fold changes in gonadotropin levels. This involved comparing the concentrations of LH and FSH in last-night-voided (LNV) and first-morning-voided (FMV) urine samples, conceptually analogous to the invasive gonadotropin-releasing hormone (GnRH) stimulation test setting. An overnight increase (the FMV/LNV ratio) in U-LH concentrations remaining at or below the cut-off level of 4.11 among girls with a U-LH concentration exceeding the cut-off level of 0.33 IU/L was suggested as a good predictor of the onset of clinical puberty within one year. In our semi-longitudinal study, which utilized single FMV and LNV urine samples from each subject, we identified that an overnight increase of 24% or less in total U-LH concentrations and 21% or less in the U-LH/U-FSH ratio in early pubertal girls could be indicative of imminent menarche (20).

As observed from the aforementioned studies, the transition from nighttime to morning levels seems to provide significant information for clinicians; therefore, potential fluctuations between samples should also be taken into consideration. This is particularly important in the pediatric population, especially for prepubertal subjects with very low levels of U-LH. Additionally, during the initial weeks and months of central (hormonal) puberty onset, nocturnal stimulation of the hypothalamic-pituitary-gonadal (HPG) axis is short-lived and does not continue into the daytime. The resultant LH secretory bursts are transient. Furthermore, the transient nature of very low levels of LH immunoreactivity, coupled with short biologic and elimination half-lives (20 min and 12 h, respectively) [37,38] and tubular degradation [39], presents major challenges. Thus, the determination of only intact LH immunoreactivity (LH-ir) from a single urine sample may be far from reflecting genuine HPG activity. Besides, the detection of the initially transient surges of hypophyseal LH secretion at minute levels that occur during nocturnal sleep necessitates the capacity to identify even low levels of immunoreactivity from any traces of LH, including its degradation products, present in urine. Our recent studies indicate that by selecting appropriate assays, total LH-ir can be measured, capturing all immunoreactive forms derived from LH degradation, including its beta-subunit (LHβ), as well as the beta core fragment (LHβcf), alongside the intact LH [40,41]. This comprehensive approach aims to capture all available indicators to avoid overlooking the minuscule amounts of pituitary LH secretion that occur during nighttime sleep in the peripubertal period. In the context of observing night-to-morning changes in very low levels of U-LH-ir, clarifying the presence and pattern of sample-to-sample variation is crucial. Currently, there is no validated method to minimize the day-to-day variance or to adjust for possible variance in U-LH-ir, as there is no information in the literature about the existence or nature of any day-to-day variation among urine samples taken at around the same time on consecutive days.

Despite a long history of achievements in developing a non-invasive urinary gonadotropin test for evaluating pubertal development and associated disorders, establishing a sampling protocol to correct for potential within-subject day-to-day variability remains unaccomplished. Recognizing this data gap, we conducted a study with daytime and evening urine samples from 95 children and adolescents to determine if significant day-to-day variation exists between U-LH-ir levels in consecutive day samples from the same individuals and, if so, to explore methods to minimize or correct for this variation.

## 2. Materials and Methods

A total of 95 children and adolescents (51 boys and 44 girls, aged 5–17 years) voluntarily participated in the study, providing daytime and evening urine samples. The study prospectively enrolled participants based on these inclusion criteria: (1) Absence of diagnosed or symptomatic endocrinologic, metabolic, oncologic, or nephrologic diseases; (2) No chronic conditions such as severe malnutrition, congenital heart disease, chronic respiratory diseases, diabetes, chronic kidney disease, muscle and nervous system disorders, metabolic diseases, or immunodeficiency; (3) No recent use of medications or hormonal contraceptives potentially impacting renal function or the HPG axis. To verify adherence to these criteria, participants underwent routine urinalysis, a complete blood count, and tests for ALT, AST, total and direct bilirubin, BUN, creatinine, and thyroid function. Additionally, a physical examination was conducted for each subject, focusing on growth and puberty, after a detailed medical history was taken. Information such as name, sex, height, weight, date of birth, age at menarche for girls, and any past diagnoses or treatments were recorded at the time of recruitment, prior to making inclusion or exclusion decisions. Pubertal development assessment followed the Tanner staging system [42].

The study was conducted with the approval of the ethics committees of the Children’s Hospital, University of Helsinki, Finland, and Dokuz Eylül University Hospital, İzmir, Türkiye. Total U-LH determinations were performed at the Department of Clinical Chemistry, University of Helsinki, Finland. Other basic laboratory analyses of the subject samples were carried out at Dokuz Eylül University Hospital. Participants provided both daytime and evening urine samples for U-LH determinations over three consecutive days. Daytime samples were collected before 12 a.m. and evening samples after 6 p.m., each preserved in individual 100 mL jars at +4 °C until transport to the hospital. A portion of each sample was used for gonadotropin analysis. The urine samples were stored at +4 °C for up to 10 days before analysis.

In this research, the employed immunofluorometric assay (IFMA) is a commercially obtainable sandwich assay utilizing monoclonal antibodies (DELFIA hLH [previously LHspec], Wallac, PerkinElmer Finland Oy, Turku, Finland). This assay involves one antibody fixed to a microplate well and another tagged with a europium chelate. Both the capture and detection antibodies target the ß-subunit of LH, recognizing separate, specific epitopes [43]. The LH assay used in this study, specifically tailored to detect intact LH and LHβ while excluding human chorionic gonadotropin, also measures LHβcf, as demonstrated in our prior research [41]. In this study, the LH assay measured total urinary LH immunoreactivity (U-LH-ir), encompassing intact LH, LHβ, and LHβcf. The assays were executed following the manufacturer’s guidelines. For both serum and urine, a sample volume of 25 µL was utilized, with the total assay volume reaching 225 µL. Calibration of the assay was against the WHO Second International Standard for human pituitary LH (80/552). The detection limit for the U-LH assay was determined at 0.015 IU/L, calculated from the measured limits of both the blank and replicates of a low-concentration analyte sample [44]. 

Batch-to-batch stability was provided by ensuring the consistency between different batches of reagents and assay components. The intra-assay and inter-assay mean coefficients of variation (CV%) for the U-LH assay were 5.2% and 6.4%, respectively [26]. For the U-LH assay, the intra-assay CV% was less than 2% for LH levels ranging from 3 to 250 IU/L and around 10% at a level of 0.3 IU/L. The inter-assay CV% for the U-LH assay was under 3% at concentrations between 5 and 18 IU/L [24]. The inter-assay CV% calculated from different daytime and evening samples of the same subject collected on three consecutive days was used as a gross measure of individual day-to-day variation in U-LH-ir (gross CV%). The net individual day-to-day variation in U-LH-ir (net CV%) was calculated by subtracting the inter-assay CV% of the assay (which is based on measurements from the same sample) from the inter-assay CV% calculated from different daytime and evening samples of the same subject on three consecutive days. Separate net CV% values were calculated for different ranges of U-LH concentrations (0.01–0.99, 1.00–1.99, 2.00–4.99, and >5.00 IU/L). As a general guideline, to assess the overall reliability of an immunoassay performed on the same sample on different occasions, the inter-assay CV% should be less than 15%.

Hormone concentration measurements were not adjusted for variations in urinary excretion rate using urine density. This decision was made because the correlation with serum levels did not show improvement with such correction, and in fact, there was an observed impairment in accuracy due to overcorrection in very dilute urine samples [14]. 

The Mauchly’s test of sphericity and repeated-measures ANOVA tests were used to analyze the within-subject variability between the daytime U-LH-ir levels measured on three consecutive days. The Greenhouse-Geisser correction was used when sphericity was shown to be violated (i.e., when *p <* 0.05). Multiple runs of the above test were performed to confirm or reject the effect of sex, age, Tanner stage, or a combination of these, on the statistical power. The Friedman’s test was used as the nonparametric alternative to repeated-measures ANOVA to compare the means of the daytime U-LH-ir levels measured on three consecutive days in small subgroups such as different Tanner and age groups, where the same subjects are represented in each subgroup and on each consecutive day. The Wilcoxon signed-rank test was used to analyze the significance of the change between different combinations of two dependent sets of laboratory results obtained on two or three consecutive days from the same subject. The design of this study was exploratory, aiming to generate new hypotheses; hence, formal power calculations were not conducted. Differences were deemed statistically significant at a *p*-value of less than 0.05.

## 3. Results

### 3.1. Day-to-Day Variation in U-LH-ir Levels in Paired Consecutive Daytime or Evening Urine Samples

The Wilcoxon signed-rank test indicated no statistically significant differences in the median urinary LH immunoreactivity (U-LH-ir) levels for most paired daytime and evening urine samples collected over three consecutive days. However, this was not the case for certain random daytime and evening urine sample pairs (Table 1). No association with sex was observed in this difference.

### 3.2. General Consistency Pattern of Individual Changes in U-LH-ir Levels over Repeated Measurements across Three Consecutive Days

In boys, the mean daytime U-LH-ir levels were not significantly different among the three time points according to the results obtained by repeated-measures ANOVA tests (F[1.541, 80.155] = 3.350, *p =* 0.052). A post hoc pairwise comparison using the Bonferroni correction showed that the difference between the mean daytime U-LH-ir levels measured on days 1 and 2 (3.0 vs. 2.0 IU/L, respectively) was not statistically significant (*p =* 0.350). The difference between the mean daytime U-LH-ir levels measured on days 2 and 3 (2.0 vs. 1.8 IU/L, respectively) was also not statistically significant (*p =* 0.999). However, the difference between the mean daytime U-LH-ir levels measured on days 1 and 3 (3.0 vs. 1.8 IU/L, respectively) was statistically significant (*p =* 0.029).

In girls, the mean daytime U-LH-ir levels were not significantly different among the three time points (F[2, 92] = 2.397, *p =* 0.097), according to the results of repeated-measures ANOVA tests. A post hoc pairwise comparison using the Bonferroni correction showed that the difference between the mean daytime U-LH-ir levels measured on days 1 and 2 (3.1 vs. 2.8 IU/L, respectively) was not statistically significant (*p =* 0.999). Similarly, the differences between the mean daytime U-LH-ir levels measured on days 2 and 3 (2.8 vs. 2.2 IU/L, respectively) and on days 1 and 3 (3.1 vs 2.2 IU/L, respectively) were not statistically significant (*p =* 0.659 and *p =* 0.059, respectively).

The mean evening U-LH-ir levels in boys were not significantly different among the three time points according to the results of repeated-measures ANOVA tests (F[1.501, 114.083] = 3.119, *p =* 0.062). Post hoc pairwise comparisons using the Bonferroni correction also showed that the differences between the mean evening U-LH-ir levels measured on days 1 and 2 (2.5 vs. 1.9 IU/L) as well as those measured on days 2 and 3 (1.9 vs. 1.5 IU/L) and on days 1 and 3 (2.5 vs. 1.5 IU/L) were not statistically significant (*p =* 0.645, *p =* 0.380, and *p =* 0.077, respectively). In girls, the mean evening U-LH-ir levels were not significantly different among the three time points (F[2, 92] = 0.419, *p =* 0.659), according to the results of repeated-measures ANOVA tests. Confirming this, post hoc pairwise comparisons using the Bonferroni correction showed that the differences between the mean evening U-LH-ir levels measured on days 1 and 2 (2.5 vs. 1.9 IU/L), between those measured on days 2 and 3 (2.9 vs. 2.5 IU/L), and between those measured on days 1 and 3 (3.0 vs. 2.5 IU/L) were not statistically significant (*p =* 0.999 for all).

Daily within-subject variability in daytime U-LH-ir and evening U-LH-ir levels determined over a period of three consecutive days was not associated with sex (F[1.795] = 0.406, *p =* 0.645, and F[1.708] = 0.471, *p =* 0.595, respectively). On the other hand, the daily within-subject variability in the mean daytime U-LH-ir from the same subjects determined over a period of three consecutive days appeared to be associated with age in a pooled population of boys and girls (F[10.805] = 2.669, *p =* 0.004); however, such an association of age with the daily variations in evening U-LH-ir levels determined over three consecutive days was not observed (F[10.283] = 0.357, *p =* 966).

As a post-hoc analysis, all the subjects were categorized into three groups of five age groups in the form of 3–7, 8–12, and 13–17 years of age. We observed that the within-subject variability of daytime U-LH-ir levels over three consecutive days may be influenced by belonging to one of the above-mentioned age groups (*p <* 0.001), but this was not true for the within-subject variability of evening U-LH-ir levels over three consecutive days (*p =* 0.629). The within-subject variability of the mean daytime U-LH-ir levels over three consecutive days was not significant in the 3–7- and 8–12-year age groups (F[1.567, 9.401] = 2.415, *p =* 0.148 and F[2, 70] = 1.449, *p =* 0.242, respectively), but was significant in the 13–17-year age group (F[2, 102] = 16.541, *p <* 0.001). All these results indicating the presence or absence of a difference between daytime U-LH-ir levels from three consecutive daytime samples were confirmed by pairwise comparisons for days 1 vs. 2, days 2 vs. 3, and days 1 vs. 3. Of these, the presence of within-subject variability in daytime U-LH-ir levels between days 1 and 2 and between days 2 and 3 among 13–17-year-old adolescents was significant (*p <* 0.001 for both pairwise comparisons). Finally, neither the mean daytime U-LH-ir nor the mean evening U-LH-ir values from the same individuals obtained by repeated daily measurements over a period of three consecutive days were affected by subject sex within any of the individual age groups mentioned above (*p =* 0.999, *p =* 0.947, and *p =* 0.414, respectively, for daytime U-LH-ir, and *p =* 0.503, *p =* 0.705, and *p =* 0.710, respectively, for evening U-LH-ir).

We further focused on analyzing the data from age groups with narrower ranges (5–6 yr, 7–8 yr, 9–10 yr, 11–12 yr, 13–14, and 15–17 yr) in order to see at what age range the within-subject variability in daytime U-LH concentrations over three consecutive days may have led to statistically significant levels. Indeed, the post hoc pairwise comparisons using the Bonferroni correction method confirmed that the within-subject variability in daytime U-LH-ir levels over three consecutive days could be influenced by belonging to one of the above-mentioned age groups (*p =* 0.004), but this was not true for the within-subject variability in evening U-LH-ir levels over three consecutive days (*p =* 0.977). The distribution of these findings, categorized by specific age groups, is summarized in Table 2. Further, subject sex was found to have no effect on the within-subject variability of daytime or evening U-LH-ir levels over three consecutive days in individual age groups of 3–4 yr, 5–6 yr, 7–8 yr, 9–10 yr, 11–12 yr, 13–14 and 15–17 yr (*p =* 0.999, *p =* 0.825, *p =* 0.706, *p =* 0.855, *p =* 0.476 and *p =* 0.408, respectively, for daytime U-LH-ir and *p =* 0.084, *p =* 0.074, *p =* 0.428, *p =* 0.758, *p =* 0.733 and *p =* 0.855, respectively, for evening U-LH-ir).

The within-subject variability of mean daytime U-LH-ir levels across the three time points was not significant within the 5–6 yr, 7–8 yr, 9–10 yr, and 11–12 yr age groups (F[2, 6] = 0.786, *p =* 0.498; F[1.041, 4.163] = 2.060, *p =* 0.223; F[1.077, 11.846] = 1.544, *p =* 0.241; and F[2, 42] = 0.524, *p =* 0.596, respectively), whereas there was a significant within-subject variability in the 13–14 yr and 15–17 yr age groups (F[2, 30] = 5.591, *p =* 0.009 and F[1.622, 58.377] = 14.308, *p* < 0.001) (Table 3). The above-mentioned results from the 5–6 yr, 7–8 yr, 9–10 yr, and 11–12 yr age groups showing no difference in daytime U-LH-ir levels measured at the three specified time points were further confirmed by post hoc pairwise comparisons with Bonferroni correction for days 1 vs. 2, days 2 vs. 3, and days 1 vs. 3. On the other hand, a significant within-subject variability in mean daytime U-LH-ir levels from day 1 to day 2 was found in the 13–14 yr and 15–17 yr adolescents (*p =* 0.009 and *p =* 0.004, respectively). In addition, significant within-subject variability was found between mean daytime U-LH-ir levels on day 1 and day 3 in the 15–17 yr adolescents (*p <* 0.001).

### 3.3. Day-to-Day Variations in U-LH-ir Levels across Different Age Groups in Consecutive Daytime and Evening Urine Samples

To confirm or discard the above exceptional result for 13–14 yr and 15–17 yr adolescents, the Wilcoxon signed-rank test was utilized to ascertain the presence of any statistically significant differences in median urinary LH immunoreactivity (U-LH-ir) levels between paired daytime and evening urine samples. These samples were randomly collected from subjects of specific age groups over three consecutive days. The age groups were formed regardless of sex because the day-to-day variation detected between the U-LH-ir levels in some paired daytime or evening samples was found to have no association with sex (Table 1). The statistical test revealed, similarly to the repeated measures ANOVA test stratified for age groups, that the mean U-LH-ir levels were significantly different between some of the two given days of the three consecutive daytime samples only in the older (13–17-year-old) adolescents but not in the relatively younger (5–12-year-old) children (Table 4). Again, no significant variability was found between the U-LH-ir levels recorded on any of the two given days out of the three consecutive evening samples in any age group in either sex.

### 3.4. Individual Changes in U-LH-ir Levels across Repeated Measurements over Three Consecutive Days

The net inter-assay CV% calculated for individual changes in U-LH-ir values over repeated measurements on three consecutive days was 24.6% and 27.3% on average in daytime samples and 28.0% to 21.6% in evening samples in boys and girls, respectively (Table 5). The level of CV% was not associated with sex, the time of urine collection (daytime or evening), or the U-LH concentration level.

## 4. Discussion

This is the first study, to the best of our knowledge, to report day-to-day variations in total U-LH-ir levels in daytime and evening urine samples taken on consecutive days from subjects in the pediatric age range. Our study highlights the importance of creating standardized urine sampling protocols to achieve consistent, clinically useful U-LH-ir test results, particularly given the lack of existing literature on the presence and characteristics of such daily fluctuations in total U-LH-ir levels.

The repeated-measures ANOVA test results showed no consistent differences in U-LH-ir levels across three consecutive sampling points for each subject, suggesting that the day-to-day variations did not follow a uniform pattern with no association to sex. Significant variations were detected in only a random subset of paired daytime or evening samples. However, the mean net within-subject CV% in three consecutive samples was found to be moderately high, ranging from approximately 17% to 34%. The inconsistency observed might result either from a random pattern of within-subject variation across three consecutive days or from the insufficient number of measurements taken, with only three time points for each subject, given that accurate CV% calculations typically require a minimum of 10, preferably 20, measurements as the established rule of thumb in laboratory practice, and ideally 50 or more [45], to ensure precision. Additionally, group comparison methods could mask individual variations when opposite changes across subjects negate each other, resulting in a seemingly stable profile for broader subpopulations but not reflecting true individual fluctuations.

Significant variation in U-LH-ir levels was observed in older adolescents (13–17 years), possibly due to their less stringent adherence to the sampling protocol, in contrast to younger subjects, whose samples were more reliably collected by parents or guardians. This might explain the paradoxical findings observed. Typically, a higher CV% would be expected in younger subjects with lower baseline U-LH-ir levels; however, we observed significant variation in the older age groups, who had higher U-LH-ir levels.

The high CV% observed in U-LH-ir levels highlights the need for more structured sampling protocols at times, such as immediately upon waking, before metabolic activity begins to vary due to food intake or daily activities, aligning with the concept of the first-morning void (FMV). Upon reviewing the outcomes of this study, it becomes apparent that our methodology, involving the collection of daytime and evening samples before 12 am and after 6 pm respectively, diverged from the ideal practice of synchronizing with periods of metabolic stability. Such a deviation presented a considerable limitation, as it permitted metabolic fluctuations to potentially influence the results. Future research should address this by incorporating more standardized timing in sample collection and expanding the number of samples to confirm the observed variability. Further investigation is also required to understand the higher variation in daily U-LH-ir levels, particularly among the older adolescent cohort.

We propose the establishment of a standardized, scheduled, and closely monitored protocol for collecting FMV and LNV urine specimens. Based on extensive data from previous research studies, it is advisable for the protocol to recommend that samples be collected no later than 9:00 a.m. for FMV urine and no earlier than 9:00 p.m. for LNV urine, consistently across at least three consecutive days. Also, strict adherence to this protocol is crucial, especially for adolescent patients aged 13 and older. Therefore, parental involvement should be a prerequisite for the protocol across all age groups. This aspect can be communicated to the older adolescents using language appropriate to their understanding, emphasizing that it is a general requirement of the study protocol and should not be taken personally. Some adolescents older than 13 years may be reluctant to cooperate. However, explaining that this is a noninvasive alternative to a blood test should encourage their participation.

Regarding the assessment of multiple test results from consecutive day samples, various approaches may be utilized. For LNV gonadotropin determinations, the best practice may involve using the average of the two closest values from the three consecutive evening results. On the other hand, the optimal approach for FMV urine samples, whether to use the average of the two closest morning values or the highest value of multiple results to better reflect HPG activity during sleep, remains to be determined. Clinicians may choose either approach, depending significantly on the specifics of each individual case, as HPG activity may not emerge consistently each night during the initial phases of central pubertal activation. In one case, a single pubertal level detected in multiple FMV U-LH determinations may serve as an indication to avoid a premature diagnosis of delayed puberty and to instead opt for a repeat series of FMV U-LH tests in due course; and in another, for instance, for a suspected precocious puberty, a clinician may require an early morning serum LH determination or a GnRH stimulation test based on the average of the two closest values. Utilizing multiple test results from consecutive day urine samples serves as a compromise alternative to the previously mentioned invasive methods. Particularly in elective cases with particularly strong reasons to avoid invasive methods, collecting multiple FMV or LNV samples at the specified times over consecutive days or weeks would provide the most definitive assessment before using established, invasive diagnostic methods.

## 5. Conclusions

For accurate urinary gonadotropin measurements in assessing pubertal development and related disorders, we emphasize the need for a standardized, scheduled, and closely monitored protocol for collecting FMV and LNV urine specimens. This approach is preferred over random daytime or evening samples to minimize errors from day-to-day variability. The approach to analyzing FMV urine samples, whether it involves averaging the two closest morning values or selecting the highest value from multiple results for a more accurate reflection of HPG activity during sleep, is at the discretion of the clinician. This decision may be based on the unique clinical aspects of each case, especially considering that HPG activity may not consistently occur each night in the initial phases of central pubertal activation. Until a reliable sampling protocol or correction method for varying urine composition is established, collecting multiple FMV or LNV samples over at least three consecutive days is advised as a temporary measure.

## Figures and Tables

**Table 1:** Statistical data on the difference in urinary luteinizing hormone immunoreactivity hormone (U-LH-ir) between any two out of three consecutive daytime and respective evening urine samples collected from the same subjects—reported separately for boys and girls. Statistically significant findings are highlighted with bold numbers.

**BOYS**	**H_0_: No Variability between Two out of Three Consecutive Days**		
**Sample Pairs**	***Md*** **_1_** **/*Md*_2_**	**Z-Statistic**	***p*** **-Value**
	(51 boys)		
**Daytime 1–2**	2.31/0.67	−2.794	**0.005**
Daytime 2–3	0.67/1.56	−0.987	0.324
**Daytime 1–3**	2.31/1.56	−3.432	**<0.001**
Evening 1–2	1.02/0.66	−0.552	0.581
Evening 2–3	0.66/0.83	−1.810	0.070
**Evening 1–3**	1.02/0.83	−2.102	**0.036**
**GIRLS**	**H_0_: No Variability between Two Out of Three Consecutive Days**		
**Sample Pairs**	***Md*** **_1_** **/*Md*_2_**	**Z-Statistic**	***p*** **-Value**
	(44 girls)		
Daytime 1–2	2.39/3.35	−2.367	0.317
Daytime 2–3	3.35/1.59	−0.697	0.742
**Daytime 1–3**	2.39/1.59	−2.665	**0.008**
Evening 1–2	2.25/1.92	−1.795	0.073
**Evening 2–3**	1.92/1.08	−2.030	**0.042**
Evening 1–3	2.25/1.08	−0.228	0.819

**Table 2:** Statistical data on the difference in urinary luteinizing hormone immunoreactivity hormone (U-LH-ir) between any two out of three consecutive daytime and respective evening urine samples collected from the same subjects—reported separately for daytime and evening urine samples. Statistically significant findings are highlighted with bold numbers.

**DAYTIME**		**H_0_: No Variability between Two out of Three Consecutive Days**		
**Age Group**	***n***	**Daytime 1 vs. 2**	**Daytime 2 vs. 3**	**Daytime 1 vs. 3**
		(Z-statistic, *p*-value)		
5–6 yr	4	1.46/0.144	−3.65/0.715	0.000/0.999
7–8 yr	5	−1.483/0.138	−1.214/0.225	−0.405/0.686
9–10 yr	12	−1.177/0.239	−0.628/0.530	−1.412/0.158
11–12 yr	22	−0.536/0.592	−0.179/0.858	−0.373/0.709
**13–14 yr**	15	**−2.386/0.017**	−0.341/0.733	−1.761/0.078
**15–17 yr**	37	**−3.176/0.001**	−0.113/0.910	**−4.005/<0.001**
**EVENING**		**H_0_: No Variability between Two Out of Three Consecutive Days**		
**Age Group**	***n***	**Evening 1 vs. 2**	**Evening 2 vs. 3**	**Evening 1 vs. 3**
		(Z-statistic, *p*-value)		
5–6 yr	4	−1.483/0.138	−0.405/0.686	−1.753/0.080
7–8 yr	5	−1.820/0.069	−0.560/0.575	−1.680/0.093
9–10 yr	12	−1.851/0.064	−0.849/0.396	−0.022/0.983
11–12 yr	22	−0.456/0.648	−1.740/0.082	−0.683/0.495
13–14 yr	15	−0.067/0.946	−0.914/0.361	−1.547/0.122
**15–17 yr**	37	−0.601/0.548	−1.569/0.117	**−2.031/0.042**

**Table 3:** Statistical data on the within-subject day-to-day variation in urinary luteinizing hormone immunoreactivity hormone (U-LH-ir) levels in three consecutive daytime and evening urine samples from the same subjects of different age groups. Statistically significant findings are highlighted with bold numbers.

**DAYTIME**		**H_0_: No Variability across Three Consecutive Days**			**H_0_: No Variability across Individual Consecutive Days**		
**Age Group**	***n***	**df**	**F**	***p*** **(Days 1, 2, 3)**	***p*** **(Days 1 vs. 2)**	***p*** **(Days 2 vs. 3)**	***p*** **(Days 1 vs. 3)**
		(51 boys, 44 girls)			(51 boys, 44 girls)		
5–6 yr	4	2, 6	0.786	0.498	0.514	0.999	0.999
7–8 yr	5	1.041, 4.163	2.060	0.223	0.644	0.690	0.999
9–10 yr	12	1.077, 11.846	1.544	0.241	0.635	0.833	0.804
11–12 yr	22	2, 42	0.524	0.559	0.999	0.999	0.999
13–14 yr	15	2, 30	5.591	**0.009**	**0.009**	0.999	0.128
15–17 yr	37	1.622, 58.377	14.308	**<0.001**	**0.004**	0.999	**<0.001**
**EVENING**		**H0: No Variability across Three Consecutive Days**			**H0: No Variability across Individual Consecutive Days**		
**Age Group**	***n***	**df**	**F**	***p*** **(Days 1, 2, 3)**	***p*** **(Days 1 vs. 2)**	***p*** **(Days 2 vs. 3)**	***p*** **(Days 1 vs. 3)**
		(51 boys, 44 girls)			(51 boys, 44 girls)		
5–6 yr	4	2, 8	1.438	0.293	0.344	0.999	0.626
7–8 yr	5	2, 14	2.387	0.128	0.336	0.999	0.452
9–10 yr	12	1.522, 25.866	0.060	0.900	0.999	0.999	0.999
11–12 yr	22	1.569, 39.223	0.650	0.491	0.999	0.792	0.843
13–14 yr	15	1.454, 33.453	1.067	0.336	0.999	0.999	0.544
15–17 yr	37	1.721, 75.729	1.789	0.179	0.692	0.999	0.331

**Table 4:** Statistical data based on a Wilcoxon signed-rank test regarding day-to-day variation in urinary luteinizing hormone immunoreactivity hormone (U-LH-ir) levels in three consecutive daytime and evening urine samples from the same subjects of different age groups. Statistically significant findings are highlighted with bold numbers.

**MORNING**		**H_0_: No Variability between U-LH-ir Levels Recorded in Two out of Three Consecutive Days**		
**Age Groups**	***n***	**Median (M1/M2/M3)**	**Z-Statistic (M1–M2/M2–M3/M1–M3)**	***p-*** **Value (M1–M2/M2–M3/M1–M3)**
	(51 boys, 44 girls)			
5–6 yr	4	0.09/0.69/0.08	−1.461/−0.365/0.000	0.144/0.715/0.999
7–8 yr	5	0.19/0.65/0.17	−1.483/−1.214/−0.405	0.138/0.225/0.686
9–10 yr	12	0.67/0.88/1.13	−1.177/−0.628/−1.412	0.239/0.530/0.158
11–12 yr	22	1.80/1.04/1.10	−0.536/−0.179/−0.373	0.592/0.858/0.709
**13–14 yr**	15	2.52/0.84/1.18	−2.386/−0.341/−1.761	**0.017**/0.733/0.078
**15–17 yr**	37	5.16/2.31/2.20	−3.176/−0.113/−4.005	**0.001**/0.910/< **0.001**
**EVENING**		**H0: No Variability between U-LH-ir Levels Recorded in Two out of Three Consecutive Days**		
**Age Groups**	***n***	**Median (E1/E2/E3)**	**Z-statistic (E1–E2/E2–E3/E1–E3)**	***p-*** **Value (E1–E2/E2–E3/E1–E3)**
	(51 boys, 44 girls)			
5–6 yr	4	0.05/0.30/0.21	−1.483/−0.405/−1.753	0.138/0.686/0.080
7–8 yr	5	0.10/0.51/0.28	−1.820/−0.560/−1.680	0.069/0.575/0.093
9–10 yr	12	0.29/0.47/0.82	−1.851/−0.849/−0.022	0.064/0.396/0.983
11–12 yr	22	1.40/1.11/0.92	−0.456/−1.740/−0.683	0.648/0.082/0.495
13–14 yr	15	1.97/1.22/1.27	−0.067/−0.914/−1.547	0.946/0.361/0.122
15–17 yr	37	3.46/2.89/2.75	−0.601/−1.569/−2.031	0.548/0.117/0.042

**Table 5:** Statistical data based on coefficient of variation (CV%) calculations regarding individual day-to-day variations in urinary luteinizing hormone (U-LH) immunoreactivity levels at different U-LH concentrations in three consecutive daytime and evening urine samples from the same subjects.

**BOYS**	**Net Interassay CV% between U-LH-ir Levels on Three Consecutive Days**			
**U-LH (IU/L)**	***n*** **(M)**	**CV% (M)**	***n*** **(E)**	**CV% (E)**
0.00–0.99	10	34.37	21	28.53
1.00–1.99	14	28.69	10	33.04
2.00–4.99	19	19.61	13	22.18
≥5.00	8	17.33	7	30.33
Overall	51	24.64	51	28.04
**GIRLS**	**Net Interassay CV% between U-LH-ir Levels on Three Consecutive Days**			
**U-LH (IU/L)**	***n*** **(M)**	**CV% (M)**	***n*** **(E)**	**CV% (E)**
0.00–0.99	12	31.73	15	27.63
1.00–1.99	5	30.53	4	24.88
2.00–4.99	20	23.94	19	16.92
≥5.00	7	27.08	6	18.89
Overall	44	27.31	44	21.56

## Data Availability

The data presented in this study are available on request from the corresponding author. The data are not publicly available due to privacy or ethical restrictions.
